# DTX2 promotes glioma development via regulation of HLTF

**DOI:** 10.1186/s13062-023-00447-w

**Published:** 2024-01-02

**Authors:** Ren Li, Yang Chen, Biao Yang, Ziao Li, Peize Li, Yu Chen, Jiayu Li, Jianhang He, Yongqiang Wu, Yanqi Sun, Xiaogang Wang, Xiaolong Guo, Wenju Zhang, Yuanli Zhao, Geng Guo

**Affiliations:** 1 School of Public Health, Shanxi Medical University, Taiyuan, 030001 Shanxi China; 2 Department of Neurosurgery, First Hospital of Shanxi Medical University, Taiyuan, 030001 Shanxi China; 3 Department of Neurosurgery, Beijing Tiantan Hospital, Capital Medical University, Beijing, 100070 China; 4 China National Clinical Research Center for Neurological Diseases, Beijing, 100070 China; 5 Department of Emergency, First Hospital of Shanxi Medical University, Taiyuan, 030001 Shanxi China

**Keywords:** Glioma, DTX2, HLTF, Ubiquitination

## Abstract

**Background:**

Human Deltex 2 (DTX2) is a ubiquitin E3 ligase that functions as an oncogene and has been shown to participate in many human cancers. However, the role of DTX2 in glioma progression has remained obscure. In this study, we explore the mechanism underlying the function of DTX2 in glioma progression.

**Methods:**

The associations between DTX2 expression and clinical characteristics of glioma were determined by bioinformatic analysis of data from The Cancer Genome Atlas and Human Protein Atlas. The expression of DTX2 in glioma tissues was detected using immunohistochemistry and western blotting. Lentivirus-mediated gene knockdown and overexpression were used to determine the effects of DTX2 and helicase-like transcription element (HLTF) on glioma cell proliferation and migration with CCK-8, cell colony formation, transwell, and wound healing assays; flow cytometry in vitro; and animal models in vivo. The interaction of the DTX2 and HLTF proteins was verified by immunoprecipitation assay and confocal microscopy.

**Results:**

DTX2 was highly expressed in glioma samples, and this was correlated with worse overall survival. Silencing of DTX2 suppressed glioma cell viability, colony formation, and migration and induced cell apoptosis. In vitro ubiquitination assays confirmed that DTX2 could downregulate HLTF protein levels by increasing ubiquitination of the HLTF protein. We also observed that HLTF inhibited proliferation and migration of glioma cells. Subcutaneous xenografts with DTX2-overexpressing U87 cells showed significantly increased tumor volumes and weights.

**Conclusions:**

We have identified DTX2/HLTF as a new axis in the development of glioma that could serve as a prognostic or therapeutic marker.

**Graphical abstract:**

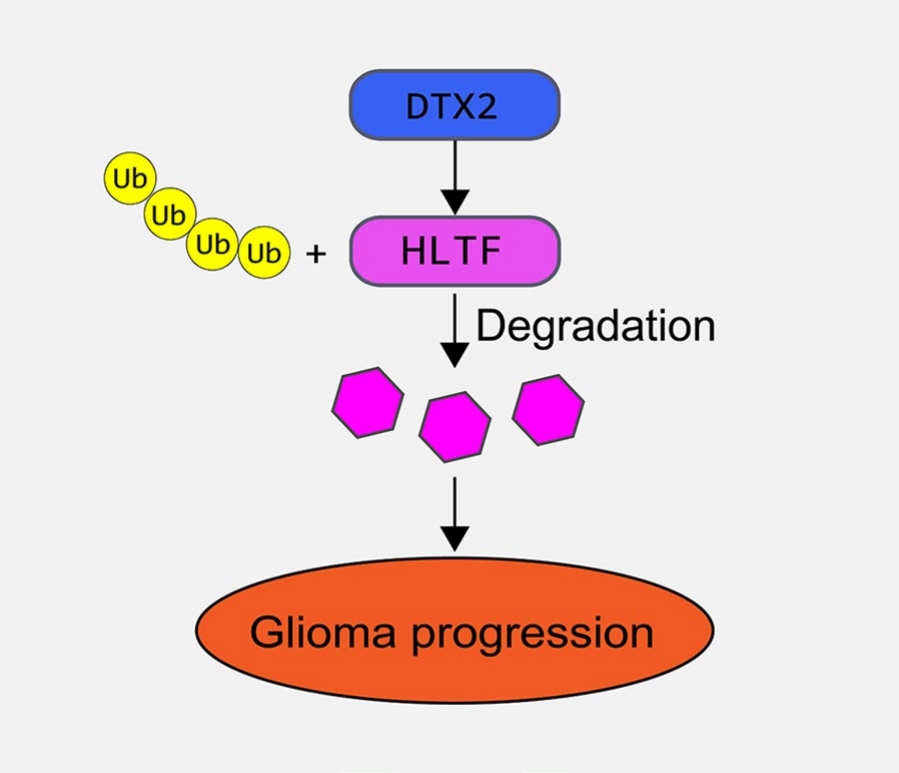

**Supplementary Information:**

The online version contains supplementary material available at 10.1186/s13062-023-00447-w.

## Instructions

Glioma consists of a group of neoplasms accounting for nearly 80% of all malignant tumors of the central nervous system [1]. The World Health Organization (WHO) classifies these into low-grade glioma (LGG) and high-grade glioma (LGG) [1]. The 5-year survival rates of patients with LGG and HGG are approximately 25% and 5%, respectively [2]. Surgical treatment, radiation, and chemotherapy are all options for the treatment of glioma; however, the curative effects of these treatments are not satisfactory. Therefore, there is a need to identify new therapeutic targets in glioma.

The human Deltex protein family of ubiquitin E3 ligases comprises five members (DTX1, DTX2, DTX3, DTX3L, and DTX4) [3, 4]. Human DTX2 on chromosome 7 (7q11.23) has been shown to be necessary for cancer cell growth. Ma et al*.* reported that DTX2 overexpression drives the motility and invasion of colorectal cancer cells via the Notch2/Akt axis [5], and Song et al*.* found that RFX6 could bind directly to DTX2 by targeting NOTCH1 in hepatocellular carcinoma, contributing to its transcriptional stability [6]. Cheng et al*.* proposed DTX2 as part of an 18-gene panel of prognostic biomarkers whose expression is related to poor outcomes in metastatic breast cancer [7]. BRD4 transcriptionally activates DTX2, contributing to glioma progression, and predicts an unfavorable prognosis [8]. However, studies of DTX2 in cancer have been limited, with very few reports on its role in glioma.

Helicase-like transcription factor (HLTF), which belongs to the SWI/SNF family, participates in tumor progression in two ways: epigenetic silencing by DNA methylation, or overexpression [9]. HLTF has been identified as a tumor-suppressive biomarker that is methylated in non-small-cell lung cancer; hypermethylation of HLTF is associated with poor survival [8]. HLTF can also be used as an independent prognostic marker of tumor recurrence [10]. However, its biological function in glioma is has remained unknown.

In this study, we demonstrate that DTX2 is overexpressed in patients with glioma and is associated with poor prognosis. DTX2 knockdown inhibits cell proliferation and induces apoptosis in glioma. We also show that higher DTX2 levels are associated with lower HLTF expression in glioma. All these findings suggest that the DTX2/HLTF axis is crucial for glioma progression and may represent a promising target for the treatment of glioma.

## Results

### High expression of DTX2 in glioma samples

**Fig. 1:**
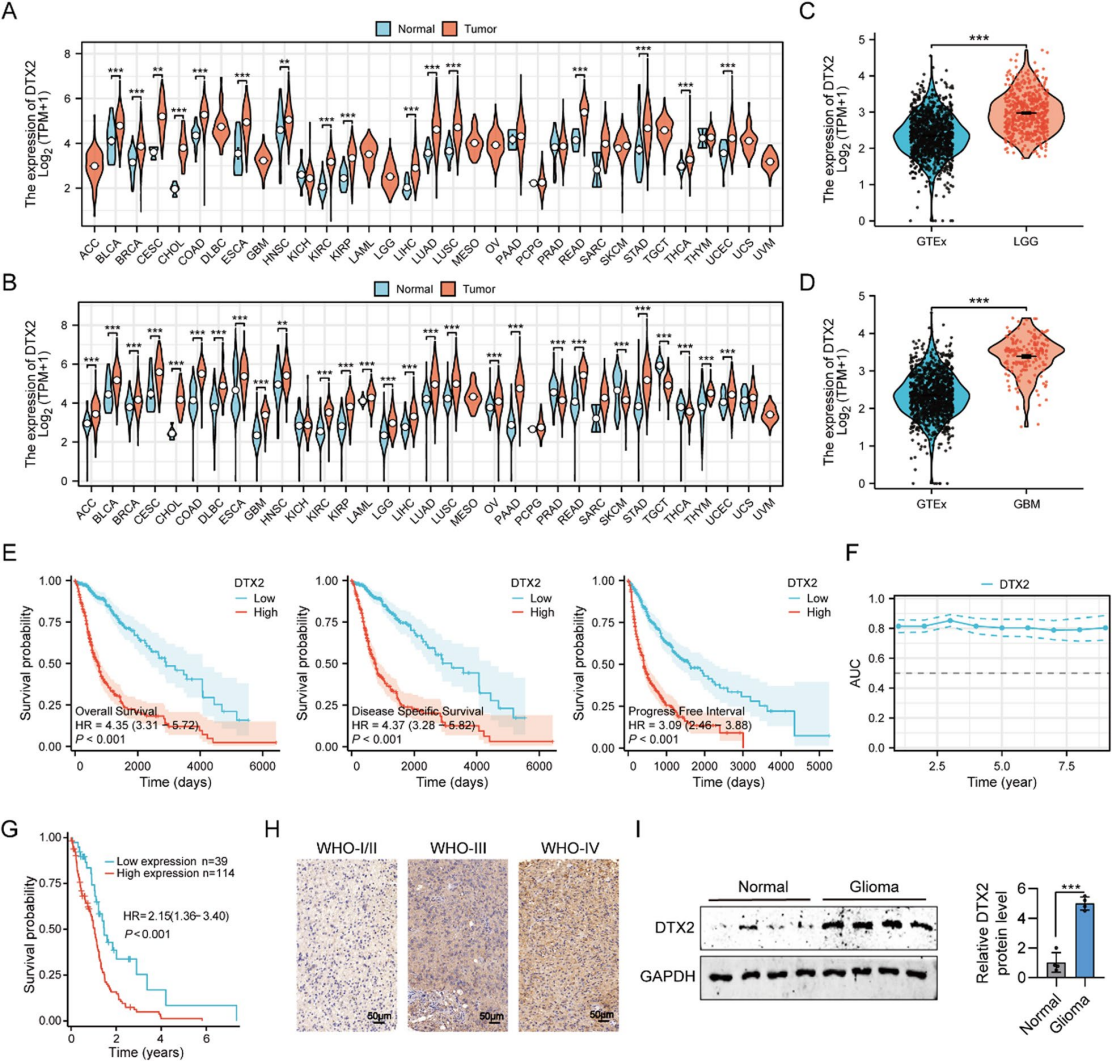
DTX2 is highly expressed and predicts poor prognosis in glioma samples. **A** Differences in mRNA levels of DTX2 in 33 cancer and paraneoplastic tissues from TCGA. **B** Differential expression of DTX2 between normal tissues from the Genotype-Tissue Expression (GTEx) database and cancer tissues from TCGA. **C**, **D** Differential expression of DTX2 in LGG (**C**) and glioblastoma (GBM) (**D**) was further analyzed in combination with normal brain tissue data from the GTEx database. Blue and red represent normal and tumor tissues, respectively. **E** DTX2 exhibited favorable prognostic value within the glioma cohort, particularly concerning OS, DSS, and PFI patterns. (Low: median =  < 50%; High: median > 50%). **F** AUC values of time-dependent ROC curves for the past decade. **G** Kaplan–Meier survival plots for glioma patients with high and low DTX2 expression from HPA. (Low: negative and weak staining; High: median and strong staining). **H** IHC staining to investigate the expression of DTX2 in clinical glioma tissues. **I** DTX2 protein expression in glioma tissues (*n* = 3) and controls (*n* = 3) as detected using western blotting

We initially analyzed the mRNA expression of DTX2 in 33 tumors of various types and corresponding paracancerous tissues obtained from The Cancer Genome Atlas (TCGA) at the pan-cancer level. The results revealed significant differences in mRNA expression of DTX2 between tumor tissues and their respective paraneoplastic tissues in 16 of the 33 analyzed tumors (Fig. 1A). Notably, DTX2 exhibited high expression specifically in tumor tissues across all identified cases. Owing to the absence of surrounding normal tissue data for certain types of cancer, we augmented our findings by integrating data from a Genotype-Tissue Expression cohort, which included normal human tissue, obtained from the XENA database. According to this complementary analysis, DTX2 exhibited significantly differential expression compared with normal tissue in 26 of the 33 tumors including LGG and glioblastomas (Fig. 1B–D).

Through a comprehensive analysis, we determined that DTX2 was closely associated with most of the unfavorable clinical features of gliomas. There was significant upregulation of DTX2 expression in glioma patients over the age of 60 years compared with younger patients (Additional file 1: Fig. S1A). The WHO grade of glioma has a strong correlation with patient survival, and our results revealed a significant increase in DTX2 expression in tumors with increasing WHO grade. Statistically significant differences were observed between the different grade groups (Additional file 1: Fig. S1B). Regarding 1p/19q co-deletion, DTX2 was highly expressed in the non-co-deletion group (Additional file 1: Fig. S1C), and it was highly expressed in the WT group with respect to IDH status (Additional file 1: Fig. S1D). DTX2 expression was highest during disease progression or the emergence of new lesions and declined when the lesions were reduced or disappeared (Additional file 1: Fig. S1E). Moreover, among the different pathological types of glioma, the expression of DTX2 was highest in glioblastoma, the most malignant type, slightly lower in astrocytoma, and lower in oligoastrocytoma and oligodendroglioma (Additional file 1: Fig. S1F). Subject characteristic curves have an important role in distinguishing clinical features of gliomas and showed high accuracy in differentiating age, WHO grade, 1p/19q co-deletion, IDH status and histological type. (Age: area under the curve (AUC) = 0.706, WHO grade: AUC = 0.846, 1p/19q co-deletion: AUC = 0.806, IDH status: AUC = 0.873, histological type: AUC = 0.845) (Additional file 1: Fig. S1G–K). However, we did not find a significant role for DTX2 in differentiating between stable and progressive stages of glioma treatment (Additional file 1: Fig. S1L). Logistic regression analysis also demonstrated a very strong association between DTX2 expression and glioma-related clinical features (Additional file 1: Fig. S1M).

Next, we conducted a prognostic correlation analysis of DTX2 using the TCGA glioma cohort; our results showed that DTX2 had favorable prognostic value within this cohort, particularly with respect to overall survival (OS), disease-specific survival (DSS), and progression-free interval (PFI) patterns (Fig. 1E). Additionally, we utilized receiver operating characteristic (ROC) curves to evaluate the discriminative ability of DTX2 in distinguishing between different clinical characteristic subtypes. The area under the ROC curve (AUC) served as the specific evaluation metric. Our result showed that DTX2 in glioma with moderate diagnostic accuracy (Fig. 1F). In addition, a prognostic nomogram was constructed by incorporating glioma clinical features and DTX2 into the prognostic model with a time reference of 1, 3, and 5 years. This showed that the higher the DTX2 expression, the lower the survival probability of patients with larger scores (Additional file 1: Fig. S2A). The calibration curves indicated that the model could provide accurate predictions of outcomes at 1, 3, and 5 years (Additional file 1: Fig. S2B). Furthermore, a Kaplan–Meier survival plot based on the HPA data clearly revealed that high DTX2 protein level was associated with poor OS of glioma patients (Fig. 1G). We also performed IHC staining to investigate the expression of DTX2 in clinical glioma tissues. DTX2 expression was significantly higher in glioma tissues of G3/G4 grade compared with those of G1/G2 grade (Fig. 1H, Table 1). Moreover, western blotting demonstrated upregulation of DTX2 protein levels in glioma tissues compared with adjacent normal controls (Fig. 1I). These findings confirm that DTX2 is highly expressed in glioma samples and correlated with worse OS.

**Table 1:** Protein abundance of DTX2 in gliomas of different grades based on IHC data

	G1–2	G3–4	χ^2^	*P* value
High expression	30	82	5.815	0.016
Low expression	29	39		
Total	59	121		

### DTX2 promotes the cell proliferation and reduced apoptosis in glioma

**Fig. 2:**
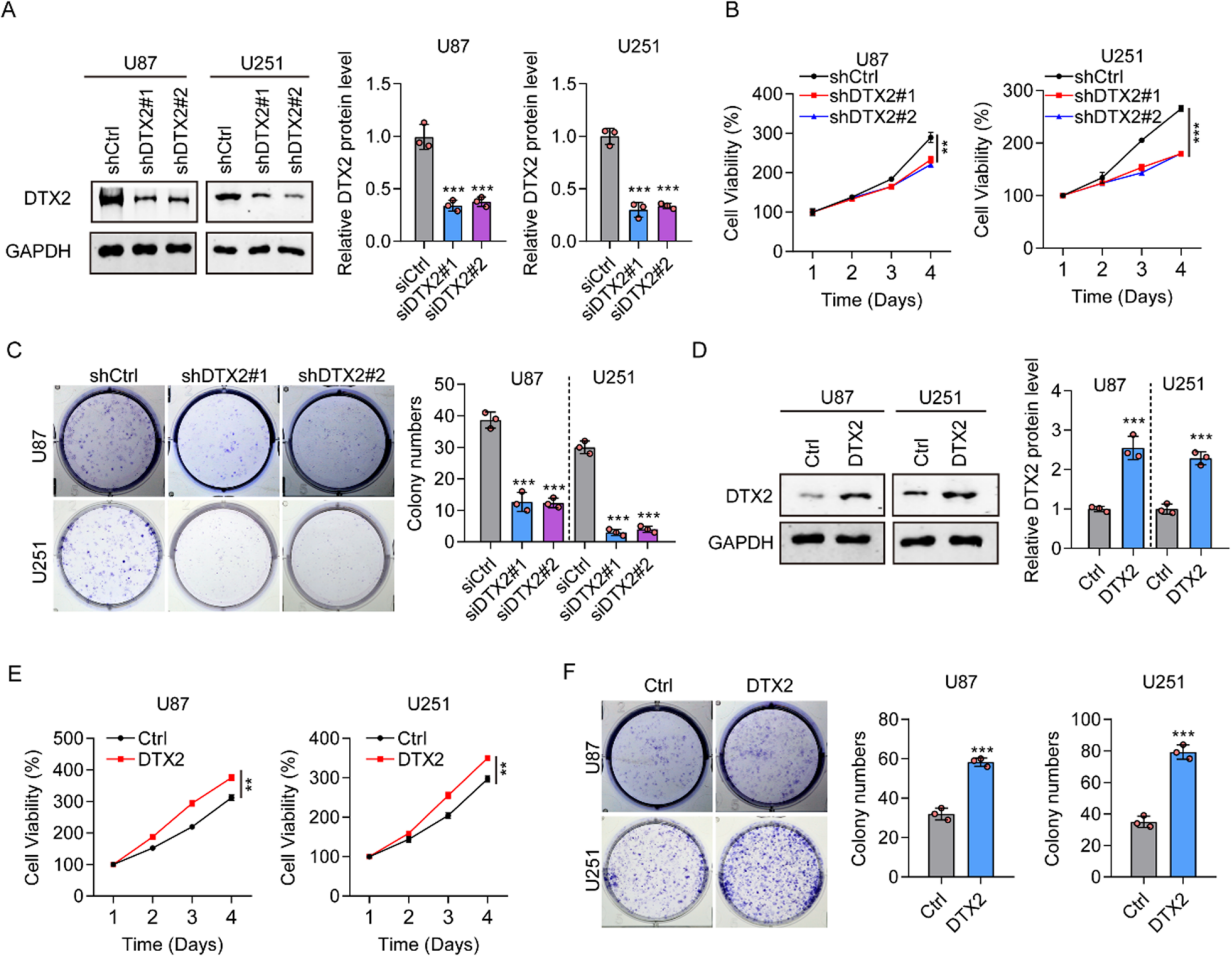
DTX2 promotes cell proliferation. **A** Western blots demonstrating the knockdown efficiency of DTX2. **B** Role of DTX2 knockdown in glioma cell propagation as determined by CCK-8 assay (**P < 0.01, ***P < 0.001). **C** Numbers of colonies formed by U87 and U251 cells transfected with shDTX2#1 and shDTX2#2 (**P < 0.01, ***P < 0.001). **D** Western blots showing the overexpression efficiency of DTX2 following transfection with overexpression plasmids. **E** Role of DTX2 overexpression in glioma cell proliferation as determined by CCK-8 assay (**P < 0.01). **F** Numbers of colonies formed by U87 and U251 cells transfected with DTX2-overexpression plasmids (**P < 0.01, ***P < 0.001)

To investigate the effects of DTX2 on the malignant behavior of glioma cells, short hairpin RNAs (shRNAs) were introduced into U87 and U251 cells to silence the expression of DTX2 (shDTX2 group), and an ectopic expression lentivirus was used to induce its overexpression. The knockdown efficiency of DTX2 was determined using western blotting (Fig. 2A). Differences in cell viability between control and DTX2-silenced glioma cells were investigated by CCK-8 assays. As shown in Fig. 2B, the cell viability of DTX2-silenced glioma cells was much lower at 72 h in both cell lines compared with controls, indicating that DTX2 repression may inhibit the development of gliomas. Furthermore, cell colony formation assays showed that U87 and U251 cells in the shDTX2#1 and shDTX2#2 groups formed fewer colonies than those in the corresponding control (shCtrl) group (Fig. 2C). The overexpression efficiency of DTX2 was determined using western blotting (Fig. 2D). Contrary to the knockdown results, DTX2-overexpressing glioma cells showed higher cell viability than the corresponding control cells (Fig. 2E). Moreover, U87 and U251 cells in the DTX2-overexpression groups formed many more colonies than those in the Ctrl group (Fig. 2F). There was an obvious increase in the proportion of DTX2-knockdown U87 and U251 cells in G0/G1 phase compared with controls (Additional file 1: Fig. S3A), and downregulation of DTX2 promoted U87 and U251 cell apoptosis (Additional file 1: Fig. S3B). Furthermore, we observed that cell apoptosis maker c-caspase3 and cell cycle maker p21 were increased in DTX2 knock down U87 and U251 cells, while BCL -2 expression was decreased in DTX2 knock down U87 and U251 cells (Additional file 1: Fig. S3C). These results indicate an oncogenic role of DTX2 in the development of gliomas.

### DTX2 promotes cell migration and invasion in glioma

**Fig. 3:**
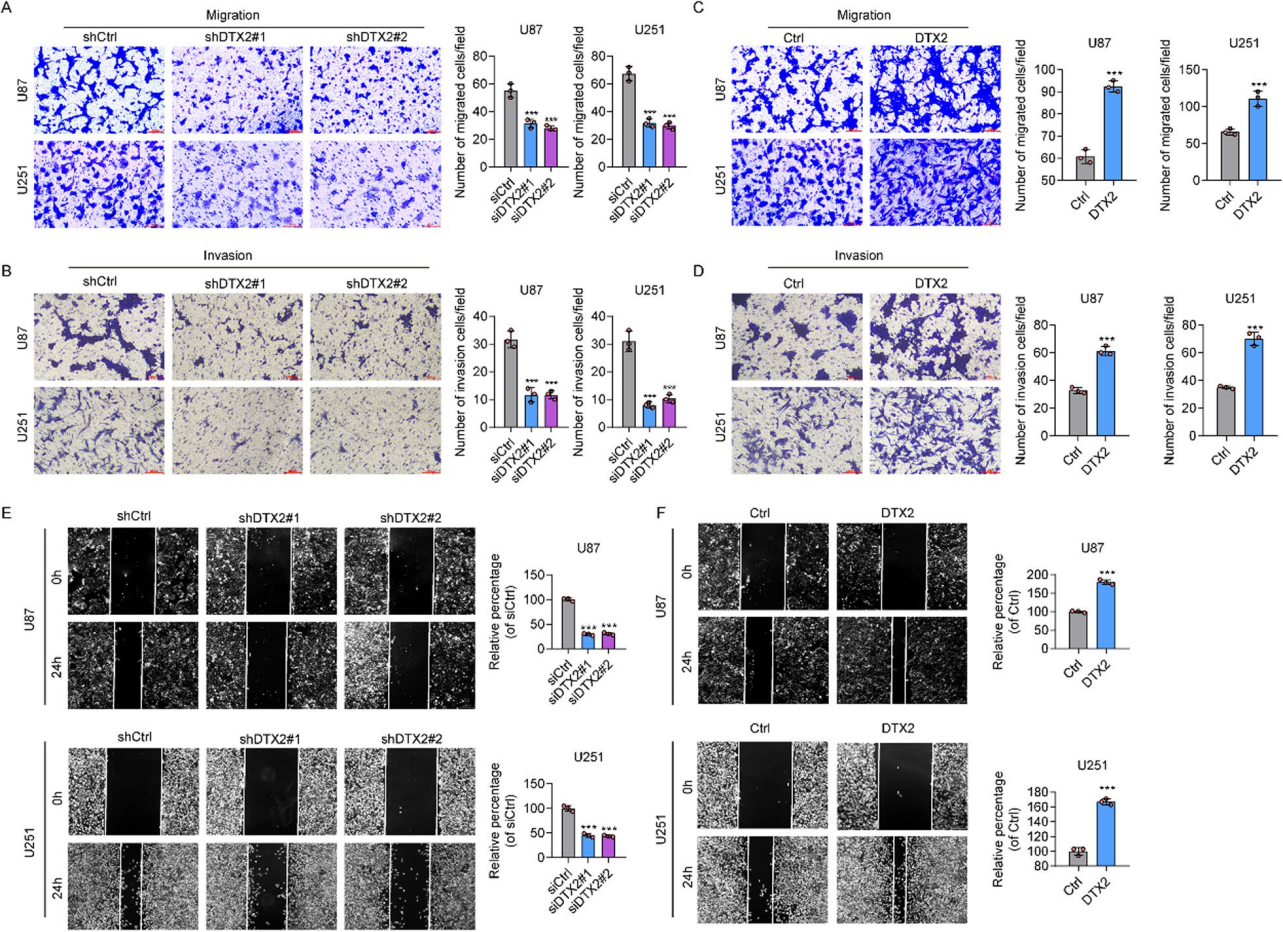
DTX2 promotes cell migration and invasion of glioma cells. **A**, **B** The role of DTX2 knockdown in the migration (**A**) and invasion (**B**) of glioma cells as determined by transwell assay (***P < 0.001). **C**, **D** Role of DTX2 overexpression in the migration (**C**) and invasion (**D**) of glioma cells as determined by transwell assay (***P < 0.001). (E, F) Wound healing assay showing cell migration of glioma cells after DTX2 knockdown (**E**) and overexpression (**F**) (***P < 0.001)

In the transwell assay, the migration and invasion of glioma cells in the shDTX2 group were greatly reduced compared with the control group (Fig. 3A, B), whereas in the overexpressed DTX2 group they were greatly increased (Fig. 3C, D). Furthermore, in wound healing assays, silencing of DTX2 was found to greatly decrease the migration distance compared with controls (Fig. 3E), whereas overexpression of DTX2 increased the migration distance (Fig. 3F). These in vitro findings suggest that DTX2 contributes to migration and invasion of glioma cells.

### DTX2 interacts with HLTF and induces its ubiquitination

**Fig. 4:**
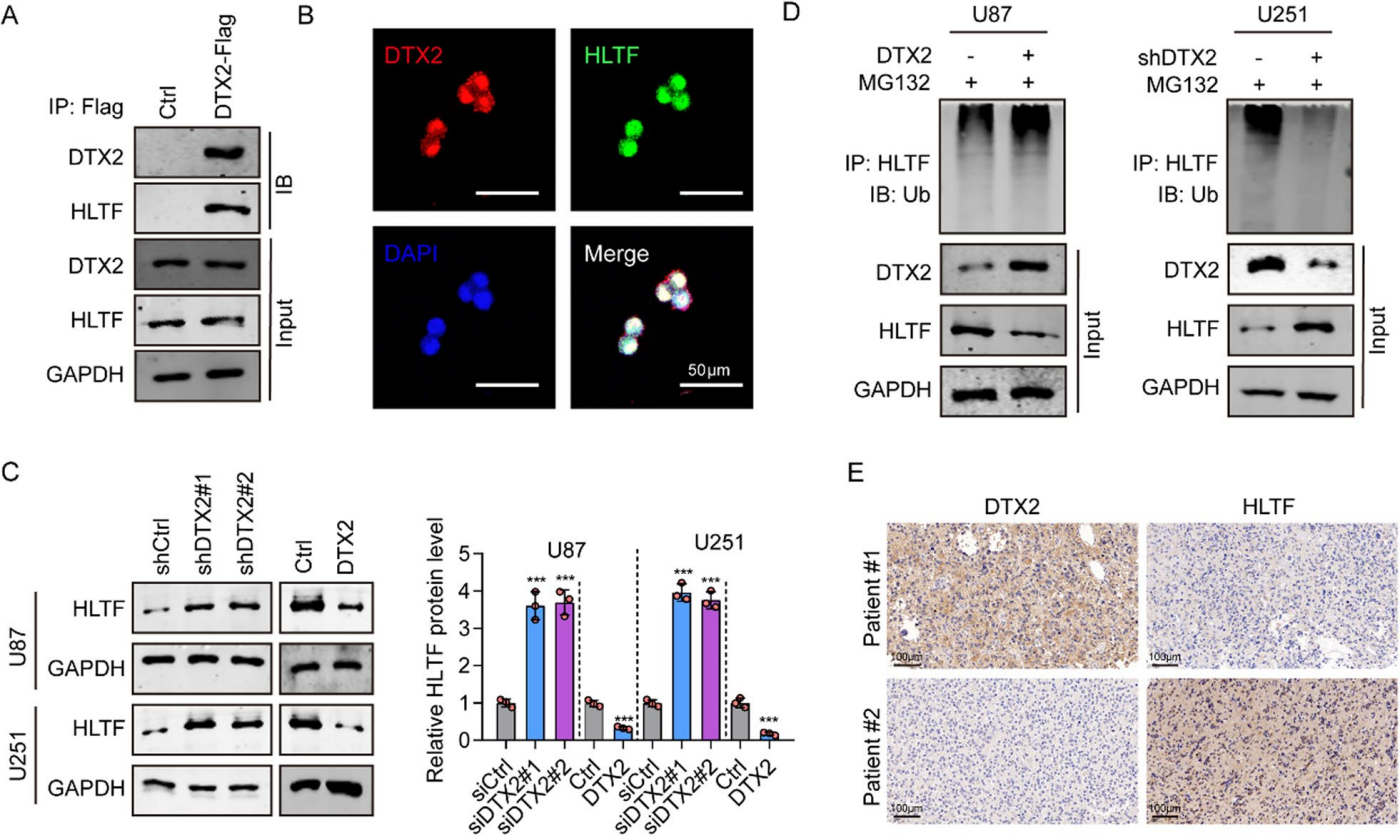
Interaction between DTX2 and HLTF. **A** HLTF protein was verified using immunoprecipitation. **B** Immunofluorescence signals showing colocalization of DTX2 and HLTF. **C** HLTF expression in glioma cells after DTX2 knockdown and overexpression as investigated by western blotting. **D** Role of DTX2 knockdown and overexpression in HLTF ubiquitination in glioma cells. **E** HLTF protein expression in glioma tissues (clinical samples)

HLTF has been proposed as a possible binding partner of DTX2 [11]. Therefore, we preformed immunoprecipitation on whole-cell lysates of human glioma cells transiently expressing Flag-tagged DTX2 or an empty vector to demonstrate complex formation. The results showed that HLTF co-immunoprecipitated with DTX2 (Fig. 4A). A previous study showed that DTX2 harbors three hidden nuclear localization signals [12]. Here, we investigated the subcellular location of DTX2 and HLTF in glioma cells was by immunofluorescence assay. The results showed DTX2 and HLTF signals co-located in the nucleus (Fig. 4B). Moreover, we used western blotting to investigate the levels of HLTF protein expression in U87 and U251 cells following DTX2 knockdown or overexpression. The results showed upregulation of HLTF expression in glioma cells with DTX2 knockdown and downregulation in the DTX2-overexpressing U87 and U251 cells (Fig. 4C), indicating a negative regulation between DTX2 and HLTF. As E3 ubiquitin-protein ligase is likely to be involved in the regulation of protein expression, we evaluated whether DTX2 regulated the stability of HLTF protein in glioma cells. In vitro ubiquitination assays confirmed that DTX2 could downregulate HLTF protein levels by increasing the ubiquitination of HLTF protein (Fig. 4D). Furthermore, we detected DTX2 and HLTF expression levels in glioma tissues by IHC and found that HLTF expression was negatively associated with DTX2 expression in 180 glioma tissues (Fig. 4E, Table 2). All of those results indicate that DTX2 binds to HLTF in glioma.

**Table 2:** Spearman correlation analysis of DTX2 and HLTF expression in 180 glioma tissues by IHC

	DTX2	
	*r*_*s*_	*P* value
HLTF	− 0.6031	< 0.01

r, Spearman correlation

### HLTF suppresses glioma cell proliferation, migration, and invasion

**Fig. 5:**
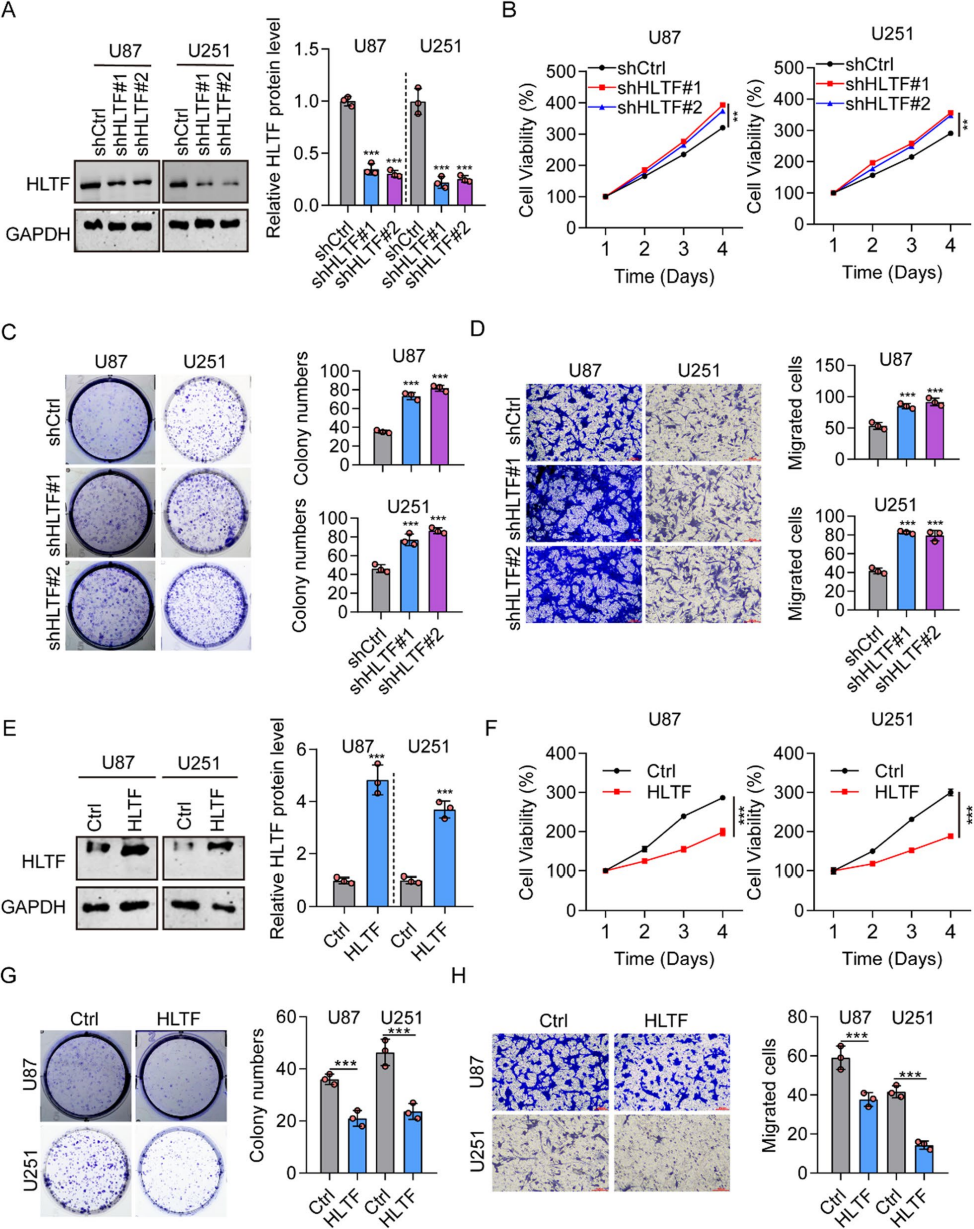
HLTF inhibits cell proliferation and migration of glioma cells. **A** The knockdown efficiency of HLTF in glioma cells was confirmed using western blotting. **B** The role of HLTF knock down in glioma cell proliferation was detected using the CCK-8 assay. (**P < 0.001). **C** Numbers of colonies formed by U87 and U251 cells transfected with shHLTF #1 and shHLTF #2. (***P < 0.001). **D** The effects of HLTF knock down in glioma cell migration were investigated using a Transwell assay. (***P < 0.001). **E** The efficiency of HLTF overexpression using HLTF overexpression plasmids in glioma cells was confirmed using western blotting. **F** The role of HLTF overexpression in glioma cell proliferation was detected using the CCK-8 assay. (***P < 0.001). **G** Numbers of colonies formed by U87 and U251 cells transfected with HLTF overexpression plasmids. (***P < 0.001). **H** The effects of HLTF overexpression in glioma cell migration were investigated using a Transwell assay. (H) The effects of HLTF in glioma cell migration were investigated using the wound healing assay

To investigate the effects of HLTF on glioma, we silenced HLTF in two glioma cell lines (U87 and U251) by RNA interference using two shRNAs (shHLTF#1 and shHLTF#2). The efficiency of HLTF knockdown was verified by western blotting (Fig. 5A). Our results showed that knockdown of HLTF stimulated cell proliferation in shHLTF#1 and shHLTF#2 glioma cells (Fig. 5B). Furthermore, cell colony formation assays showed that U87 and U251 cells in the shHLTF#1 and shHLTF#2 groups formed more colonies than those in the shCtrl group (Fig. 5C). Transwell assays showed that knockdown of HLTF increased the migration ability of U87 and U251 cells (Fig. 5D). The efficiency of HLTF overexpression was determined using western blotting (Fig. 5E). In contrast to the results for HLTF-knockdown cells, HLTF-overexpressing U87 and U251 glioma cells showed lower cell viability (Fig. 5F), formed fewer expression colonies (Fig. 5G), and had decreased migration ability (Fig. 5H) compared with controls. These results confirm the tumor suppressor role of HLTF in the development of glioma.

### DTX2 suppresses glioma cell proliferation, migration, and invasion through HLTF

**Fig. 6:**
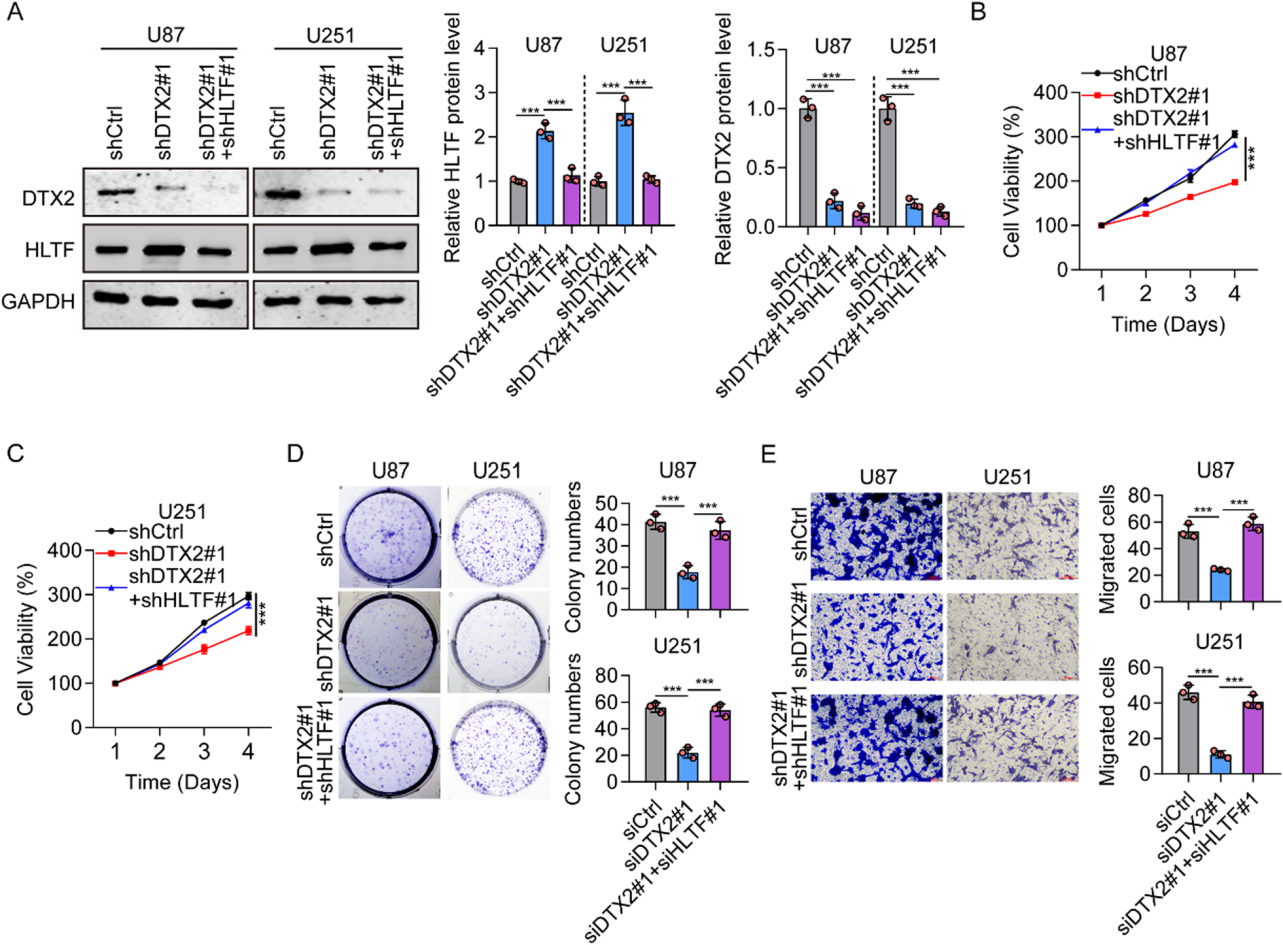
DTX2/HLTF axis contributes to glioma cell proliferation and migration through HLTF. **A** The expression of DTX2 and HLTF was detected DTX2 knock down and double knock down of DTX2 and HLTF (shDTX2#1 + shHLTF#1) in U87 and U251 cells by western blot. **B**, **C** The role of DTX2 and HLTF knock down in glioma cell proliferation was detected in U87 and U251 cells using the CCK-8 assay. (***P < 0.001). **D** Numbers of colonies formed by U87 and U251 cells with DTX2 knock down and double knock down of DTX2 and HLTF (shDTX2#1 + shHLTF#1) in U87 and U251 cells (***P < 0.001). **E** The effects of DTX2 and HLTF knock down in glioma cell migration were investigated using a Transwell assay. (***P < 0.001)

To investigate whether the effects of DTX2 on glioma depended on HLTF, we performed double knockdown of DTX2 and HLTF (shDTX2#1 + shHLTF#1) in U87 and U251 cells. Our results confirmed that expression of DTX2 was decreased in both DTX2-knockdown and double-knockdown (shDTX2#1 + shHLTF#1), whereas expression of HLTF was increased in the DTX2-knockdown cells (Fig. 6A). In addition, knockdown of DTX2 inhibited cell proliferation in both glioma cell lines (Fig. 6B, C). Cell colony formation assays showed that U87 and U251 cells in the shDTX2#1 group formed fewer colonies than those in the shCtrl group, whereas the colony-formation ability was rescued in the shDTX2#1 + shHLTF#1 group (Fig. 6D). Transwell assays showed that knockdown of DTX2 decreased the ability of migration of U87 and U251 cells; this ability was again rescued in the double-knockdown group (Fig. 6E). All those results suggest that DTX2 suppresses glioma cell proliferation, migration, and invasion through HLTF.

### DTX2/HLTF axis promotes glioma development in vivo

**Fig. 7:**
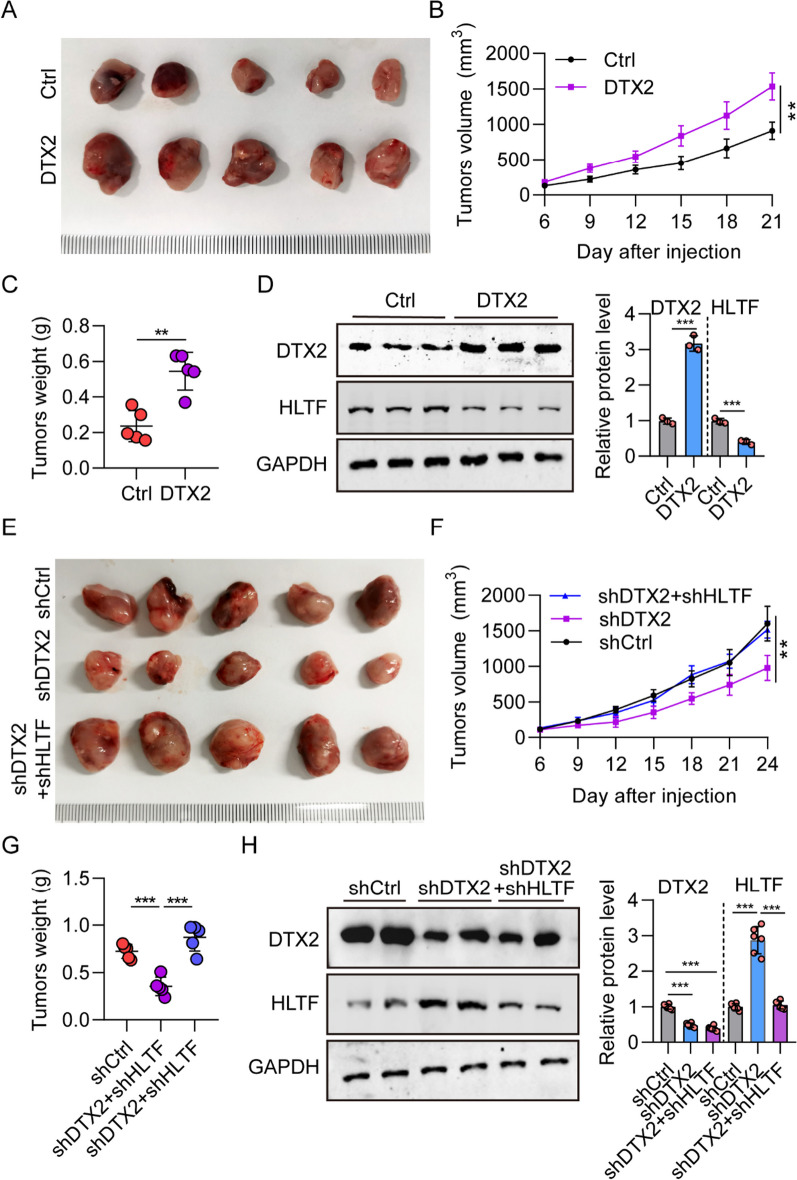
DTX2 promotes the development of glioma through HLTF in vivo. **A** Representative images of tumor size in DTX2 overexpression in the tumor xenograft model. **B**, **C** Tumor volume and tumor weight in mice xenografts in the Ctrl and DTX2 groups. (**P < 0.01). **D** The expression of DTX2 and HLTF in mice tumors in the Ctrl and DTX2 groups detected using western blot. **E** Representative images of tumor size in shCtrl, shDTX2 and shDTX2 + shHLTF xenograft models. **F**, **G** Tumor volume and tumor weight in shCtrl, shDTX2 and shDTX2 + shHLTF xenograft models. (**P < 0.01). **H** The expression of DTX2 and HLTF in mice tumors in shCtrl, shDTX2 and shDTX2 + shHLTF mice tumors detected using western blot

Having shown that DTX2 promoted the development of glioma and negatively regulated the expression of HLTF in vitro, we next investigated whether such regulation had an effect on in vivo tumor growth in nude mice models. A tumor xenograft model was established by injecting Ctrl, DTX2, shCtrl, shDTX2, DTX2, and shDTX2 + shHLTF U87 cells into the frontal lobes of nude mice. We observed that overexpression of DTX2 in U251 glioma cells greatly induced their tumorigenic capacity (Fig. 7A). Subcutaneous xenografts with DTX2-overexpressing U87 cells showed significantly increased tumor volumes (Fig. 7B) and tumor weights (Fig. 7C). We also confirmed by western blotting that the expression of DTX2 was increased in tumors with DTX2 overexpression, whereas the expression of HLTF was decreased (Fig. 7D). Furthermore, we observed that shDTX2#1 could suppress tumorigenic capacity relative to the shCtrl group, whereas this capacity was rescued in the shDTX2#1 + shHLTF#1 group (Fig. 7E–G). Finally, we confirmed by western blotting that the expression of DTX2 was decreased in tumors of the DTX2-knockdown mice, whereas it was increased in tumors in the DTX20-overexpression group (Fig. 7H). These results suggest that the DTX2/ HLTF axis promotes glioma development in vivo*.*

## Discussion

The characteristics and functions of the DTX family proteins have been investigated for more than a decade owing to their potential roles in cancer diagnosis and therapy [4]. In mammals, the DTX family of RING E3s consists of DTX1, DTX2, DTX3, DTX3L, and DTX4 [13]. The characteristics of members of this family include a conserved C terminus consisting of a RING and DTC domain [11]. DTX2 has been reported to be involved in many human cancers with functions as an oncogene and can be used as a prognostic marker [5, 8]. In this study, we showed that the high expression of DTX2 in glioma tissues was associated with poor patient prognosis. Upregulation of DTX2 drove cell proliferation, migration, and invasion, whereas engineered downregulation of DTX2 had the opposite effects, consistent with the oncogenic role of DTX2 in the development of glioma.

HLTF belongs to the SWI/SNF family of proteins that participate in chromatin remodeling. It has a RING finger motif characteristic of ubiquitin ligase proteins and acts as a tumor suppressor in cancers [14, 15]. Liu et al*.* reported a negative relationship between HLTF and the progression of colorectal cancer and found that its overexpression controlled the movement and invasion of colorectal cancer cells by targeting the TGF‑β/SMAD pathway [16]. Tan et al*.* showed that there was significant downregulation of HLTF in hepatocellular carcinoma tissues and a positive association with patient survival [17]. To elucidate the mechanisms underlying the role of DTX2 in the progression of glioma, we identified five potential downstream proteins of DTX2 described in the literature [11]. Furthermore, we found that HLTF could bind to DTX2 in glioma cells, and that the two proteins were co-located in the nucleus. Western blotting revealed that protein levels of HLTF in glioma cells were greatly reduced after overexpression of DTX2 but was highly increased after DTX2 knockdown, indicating the negative regulation of DTX2 and HLTF. Our results also showed that ectopic expression of HLTF decreased cell growth and invasion of glioma cells, whereas silencing of HLTF induced glioma cell growth and metastasis. In vitro ubiquitination assays showed increased HLTF ubiquitination in DTX2 overexpressed glioma cells and decreased HLTF ubiquitination in DTX2 knockdown cells, which confirmed our assumptions. Finally, tumor xenograft experiments further verified the involvement of the DTX2/HLTF axis in tumor growth in glioma. The limitation of this study is that the HLTF expression and prognostic value in glioma samples also need further investigating.

## Conclusions

Collectively, the results of this study demonstrated the involvement of the DTX2/HLTF axis in glioma cell progression and tumor growth. High DTX2 expression could promote glioma cell development and indicate poor prognosis of glioma patients. Furthermore, DTX2 regulated the expression of HLTF through ubiquitination. The newly identified DTX2/HLTF axis strengthens the potential oncogenic role of DTX2 and tumor suppressor role of HLTF in glioma and provides potential therapeutic markers for glioma.

## Methods and materials

### Samples from TCGA and proteomics data

Glioma data (including LGG and glioblastomas) were captured from the TCGA database (https://portal.gdc.cancer.gov/) [18]. There were 706 glioma samples available (including 532 LGG and 174 glioblastoma samples). Data for 1152 normal brain tissue samples were acquired from the UCSC XENA database (https://xenabrowser.net/datapages/) [19]. Data from glioma samples without clinical information were discarded and supplemented with data on WHO classification, IDH mutation status, and 1p/19q co-deletion from Ceccarelli et al. [20]. All subsequent analyses were performed using R software, version 4.2.3.

IHC data were acquired from HPA (https://www.proteinatlas.org/) [21] for normal tissue samples as well as LGG and glioblastomas from humans. We also obtained pertinent immunofluorescence protein images from HPA, further enhancing the comprehensiveness of our analysis. The variance results were visualized using the R package “ggplot2”.

### Clinical specimens and IHC analysis

A total of 180 glioma tissues specimens were provided by the Department of Neurosurgery at the First Hospital of Shanxi Medical University. All samples were diagnosed by two independent neuropathologists according to the WHO classification. They were diagnosed by histopathology and received no treatment prior to the operation. All patients provided their informed consent for sampling and molecular evaluation. The Ethics Committee of the First Hospital of Shanxi Medical University approved this study.

Paraffin-embedded subcutaneous xenograft specimens were cut into 5-μm sections, and IHC analysis was performed as described previously [22]. The primary antibodies used were as follows: anti-DTX2 antibody (1:200, PA5-109664, ThermoFisher), anti- HLTF (ab17984, abcam, 1:200). Immune staining was visualized with a PV-9000 polymer detection system according to the manufacturers’ instructions.

### Cell culture and lentivirus infection

U81 and U251 cell lines were obtained from our laboratory and were cultured in Dulbecco’s modified Eagle medium (DMEM) with 10% fetal bovine serum (FBS) in a humidified atmosphere of 5% CO_2_ at 37 °C. Lentivirus-mediated knockdown and overexpression were use to silence and overexpress DTX2 and HLTF in glioma cells, respectively. The shRNA sequences used were as follows: shDTX2#1, 5′-CCUCAUAGUUUACAGCAUU-3′, shDTX2#2, 5′-GGAACGAGAUCCACCACAA-3′, shHLTF#1: 5′-GCCACAUGCAAAGUGUCCUUU-3′, shHLTF#2: 5′-GCAGGUGGAGUUGGUUUGAAU-3′, shCtrl, 5′-CAGUACUUUUGUGUAGUACAAA-3′. All lentiviruses were purchased from Syngentech (Beijing, China) and used in accordance with the instructions provided.

### Real-time PCR

The TRIzol® RNA isolation reagent was used to extract total RNA from transfected U87 and U251 cells. A PrimeScript RT reagent kit was used to perform reverse transcription, and quantitative reverse transcriptase PCR was performed using Synergy (SYBR) Premix ExTaq™ on a Light Cycler 480II real-time PCR system. Normalized 2^−ΔΔCt^ expression of GAPDH, the housekeeping gene from the control group, was used to quantify relative levels of mRNA. The following primers were used: DTX2, forward, 5'-TGGCTCCTGGACTGCCTAT-3′ and reverse, 5′- GGGTGGTGTAGTTGACAGTGTA-3′. HLTF, forward, 5′- TTTTCCACGCCTCTCATATCCA-3′ and reverse, 5′-AGCGTAGTCCAACCACATGAC-3′. GAPDH, forward, 5′-TGTGGGCATCAATGGATTTGG-3′ and reverse, 5′- ACACCATGTATTCCGGGTCAAT-3′.

### Western blotting

Total protein from cells or tissues was extracted using RIPA lysate supplemented with protease inhibitor, and protein concentrations were measured using BCA assay. Samples were denatured by boiling for 5 min, and then 30 μg/well per sample was loaded onto a gel for sodium dodecyl sulfate polyacrylamide gel electrophoresis (SDS-PAGE). Following electrophoresis, the proteins were electrotransferred to a PVDF membrane, followed by blocking for 2 h with 5% bovine serum albumin at room temperature. The membranes were incubated overnight at 4 °C with the following primary antibodies: DTX2 (1:1000, PA5-109664, ThermoFisher), HLTF (ab17984, abcam, 1:1000), and GAPDH (ab263962, abcam, 1:1000), p21 (ab109520, abcam, 1:1000), Caspase3 (ab184787, abcam, 1:1000) and Cleaved Caspase3 (ab2302, abcam, 1:1000), Bcl-2 (ab241548, abcam, 1:1000). After three washing steps with TBST, a diluted secondary antibody (A16096, Invitrogen, 1:2000) was added to the membranes, followed by incubation at room temperature for 2 h. ECL chemiluminescence was used to develop protein signals, and samples were photographed. Applying GAPDH as the internal reference, the average grey densitometric value of each band was quantified using ImageJ software.

### Cell viability

Cell Counting Kit-8 (CCK-8) assay was used to measure cell viability. Briefly, in a 96-well plate, infected U87 and U251 cells were seeded at a density of 1,000 cells/well and incubated overnight. After washing with phosphate-buffered saline (PBS), fresh medium containing CCK-8 reagent was used to replace the growth medium. After 1 h of incubation, a FLUOstar Omega (BMG Labtech, Offenburg, Germany) plate reader was used to measure absorbance at 450 nm.

### Cell apoptosis assay

Flow cytometry was used to detect cell apoptosis. The infected cells were collected after 48 h of culture and stained with Annexin V-FITC and propidium iodide according to the manufacturer’s protocol. A Beckman CytoFLEX flow cytometer was used to detect fluorescence signals.

### Transwell assay

Cell invasion and migration were investigated using transwell assays. Briefly, 1.5 × 10^5^ cells were seeded in serum-free medium in the upper chamber with or without a Matrigel coating, and 500 μL of DMEM with 10% FBS as a chemoattractant was used to fill the lower chamber. After 24 h of incubation, a cotton swab was used to remove non-invading cells from the upper chamber. Cells were fixed with methanol, and the membranes were stained with 0.1% crystal violet. Migrating cells were counted and reported as mean ± standard deviation (*n* = 3).

### Wound healing assay

Infected U87 and U251 cells were cultured for 6–8 h in complete medium until cell confluence reached more than 90%. Three scratches with a sterilized 200-μl pipette tip were carefully made as a reference line in the cell monolayer, and floating cells were washed away with prewarmed PBS, avoiding disturbance of the cell layer at the edge of the scratch. The cells were then covered with FBS-free DMEM and cultured in a 37 °C incubator. The migration and healing of cells were visualized under a microscope at 0 h and 24 h and photographed.

### Co-immunoprecipitation assay

Cell lysis was carried out in immunoprecipitation buffer supplemented with protease- and phosphatase-inhibiting agents, and cells were collected by centrifugation for 10 min at 12,000 × g and 4 °C. Immunoprecipitation of resuspended protein in the supernatants was performed with antibodies following 2 h of incubation with magnetic protein A/G beads (Pierce) at 4 °C. After being washed three times with PBS, the immune complexes were resuspended in SDS-PAGE buffer and subjected to western blotting.

### Immunostaining and confocal microscopy

Briefly, cells were fixed in 4% paraformaldehyde on microscope slides and subjected to permeabilization using 0.5% TritonX-100 before incubation with primary antibodies (anti-DTX2, PA5-60,164, Invitrogen; anti-HLTF, PA5-82,525, Invitrogen) and secondary antibodies (Cat# A-11011, Cat# A-11008, Invitrogen). Nuclei were stained with Hoechst 33,342. Cell visualization was conducted under a confocal laser scanning microscope. The images were evaluated as previously described [23].

### Ubiquitination assay

DTX2-overexpressing U87 cells and DTX2-silenced U251 cells were cultured and transfected using an HA-Ub plasmid for 24 h. After treatment with MG132 (10 μM) for 4 h, cell lysis was performed in NETN buffer plus 0.05% SDS. Lysate sonication was carried out at 30% amplitude with five cycles of 2 s on and 8 s off, followed by a 10-min centrifugation at 12,000×*g* and 4 °C. Approximately 1% of the lysate was saved as the input solution. Glutathione Sepharose 4B was used to precipitate the remaining lysate. Primary antibodies were used for immunoblotting to analyze the precipitates.

### Tumor xenograft model

BALB/c mice (4 weeks old) were provided by Beijing Vital River Animal Experimental Technology Co., Ltd. Mice were housed and reared under specific-pathogen-free conditions and were randomly allocated for testing. For the subcutaneous xenograft model, a subcutaneous injection of 6 × 10^6^ Ctrl, DTX2, shCtrl, shDTX2, or and shDTX2 + shHLTF U87 cells was administered into the frontal lobes of BALB/c mice (*n* = 5 mice in each group). Tumor size was measured every 3 days, and tumor volume were calculated using the following formula: (length × width^2^)/2. Mice were euthanized at the end of the experiment. The Institutional Animal Care and Use Committee of First Hospital of Shanxi Medical University approved all animal protocols.

### Association of DTX2 with clinical features of glioma and ROC diagnostic analysis

We examined the relationship between glioma clinical features and DTX2 expression, assessing differences in DTX2 levels across various clinical subtypes. In addition, we used ROC curves to evaluate the discriminative ability of DTX2 in distinguishing between different clinical characteristic subtypes. The AUC of the ROC curve served as the specific evaluation metric: AUC values below 0.7 indicated low diagnostic accuracy, AUC values ranging from 0.7 to 0.9 indicated moderate accuracy, and AUC values exceeding 0.9 reflected high diagnostic accuracy. The Wilcoxon rank-sum test was used to compare the two groups statistically. For WHO grade and histology type analyses, Kruskal–Wallis test was used, followed by Dunn’s test. The primary therapy outcome was assessed using Welch one-way analysis of variance, with post-hoc analysis using the Games-Howell test. A *P* value < 0.05 was considered to indicate statistical significance. Logistic models for DTX2 were constructed separately for each clinical feature, and the effects of DTX2 on the clinical feature of interest were evaluated. All variables were grouped according to the median.

### Analysis of the survival value of DTX2 in glioma and construction and evaluation of the prognostic model

Patients with glioma were divided into high and low expression groups based on the median DTX2 expression value. Kaplan–Meier survival curves were used to analyze the difference in survival probability between the high and low expression groups in terms of OS, DSS, and PFI. For further analysis of the survival value of DTX2 in LGG and glioblastoma, time-dependent ROC curves were constructed to appraise the prognostic value of DTX2 in glioma. The survival curves were statistically analyzed using Cox regression, and a *P* value < 0.05 was considered statistically significant. Clinical features of glioma associated with prognosis and DTX2 expression were included in the analysis to construct a prognostic nomogram for glioma patients. All variables were categorical; histological type was included and excluded based on covariance analysis. A total of 620 glioma samples were included in the prognostic model. Prognostic calibration curves were drawn based on two samples of 80, with 200 replicates, and raw data for the DTX2 protein expression survival cohort were obtained from HPA. All survival analyses were performed using the R package “survival”.

### Statistical analysis

All values are represented as mean ± standard deviation. GraphPad 9.0 was used to analyze the data. Student’s t-test and one-way analysis of variance followed by Tukey’s post hoc test were used to compare differences between two groups and differences among three or more groups, respectively. P < 0.05 was considered to indicate statistical significance.

### Supplementary Information

**Additional file 1: **Supplementary Figures.

## Data Availability

The datasets generated and analyzed in this research are available from the corresponding author on reasonable request.

## Abbreviations

DTX2: Deltex E3 ubiquitin ligase 2
HLTF: Helicase-like transcription element
WHO: World Health Organization
LGG: Low grade glioma
HGG: High-grade glioma
AUC: The area under the curve
ROC: Receiver operating characteristic
TCGA: The Cancer Genome Atlas Project
GTEx: Genotype-Tissue Expression database
GBM: Glioblastoma
