# Supplementary material for: DTX2 promotes glioma development via regulation of HLTF
Source: Biol Direct. 2024 Jan 2;19:2. doi: 10.1186/s13062-023-00447-w (PMC10759338; doi:10.1186/s13062-023-00447-w)
Supplement: Supplementary file 1 — Additional file 1: Supplementary Figures. [file 13062_2023_447_MOESM1_ESM.docx]

**Figure S1.**


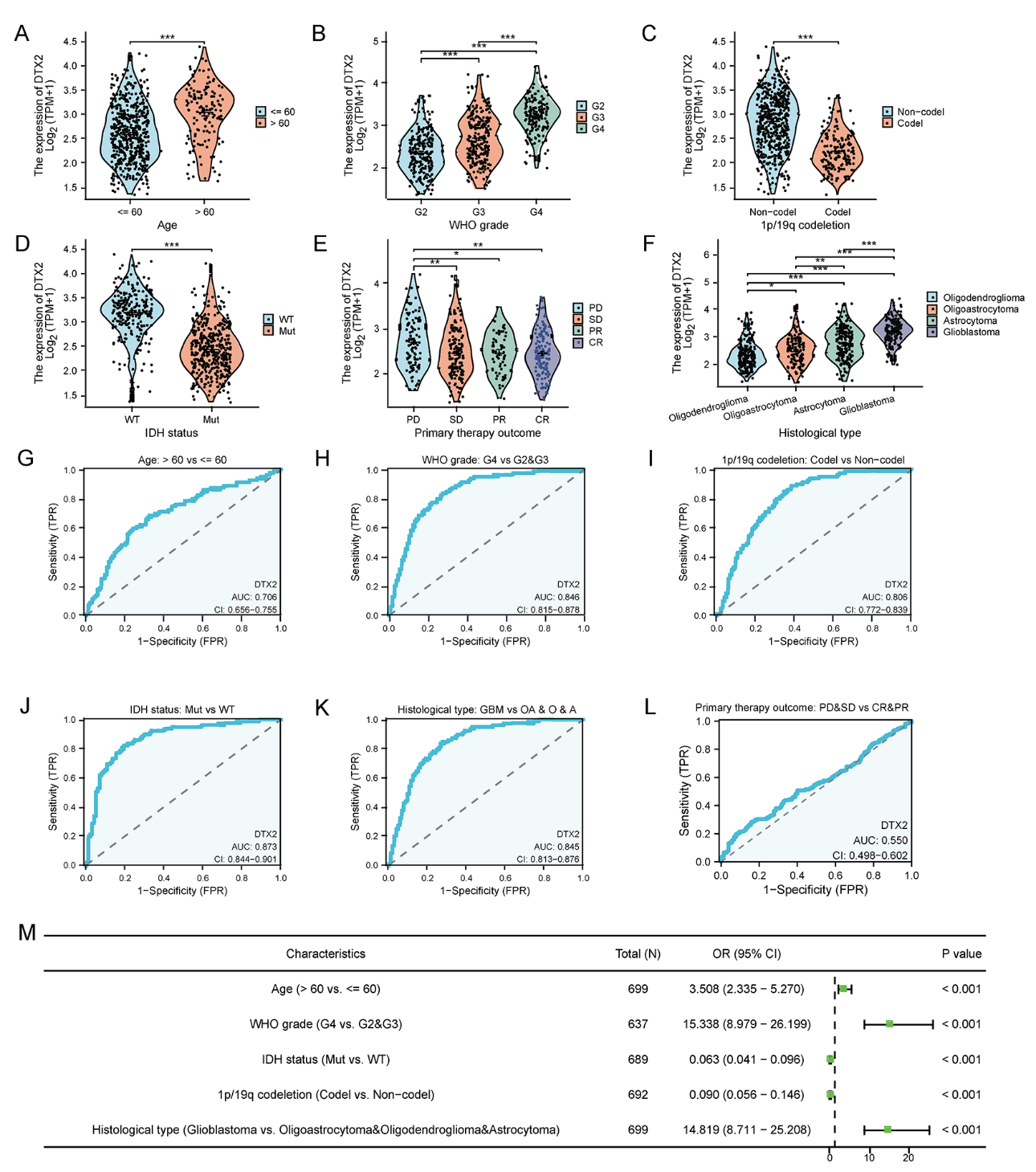


**Figure S1. High DTX2 expression was associated with adverse clinical features associated with glioma.** (A) DTX2 was highly expressed in glioma patients aged >60 years. (B) DTX2 expression was highest in WHO G4 and lowest in G2. (C) In the co-del group in the 1p/19q co-deletion, DTX2 is lowly expressed. (D) DTX2 expression was lower in IDH status mutants. (E) DTX2 levels were highest during lesion progression and lower during complete and partial disease remission. (F) Among the different pathological subtypes of glioma, the highest DTX2 levels were found in glioblastoma, followed by astrocytoma. (G-L) ROC diagnostic curves to investigate the diagnostic efficacy of DTX2 in clinical features associated with glioma patients, including age, WHO grade, IDH status, 1p/19q codeletion, primary therapy outcome and histological subtype. (M) Single-gene logistic regression models further assessed the effect of DTX2 on the analysis of clinical variables and showed that DTX2 had a relatively significant effect on the analysis of all clinical indicators of glioma. Remark: PD, Progressive disease; SD, Stable disease; PR, Partial response; CR, Complete response; O, Oligoastrocytoma; A, Astrocytoma; OA, Oligodendroglioma; GBM, Glioblastoma.

**Figure S2.**

**
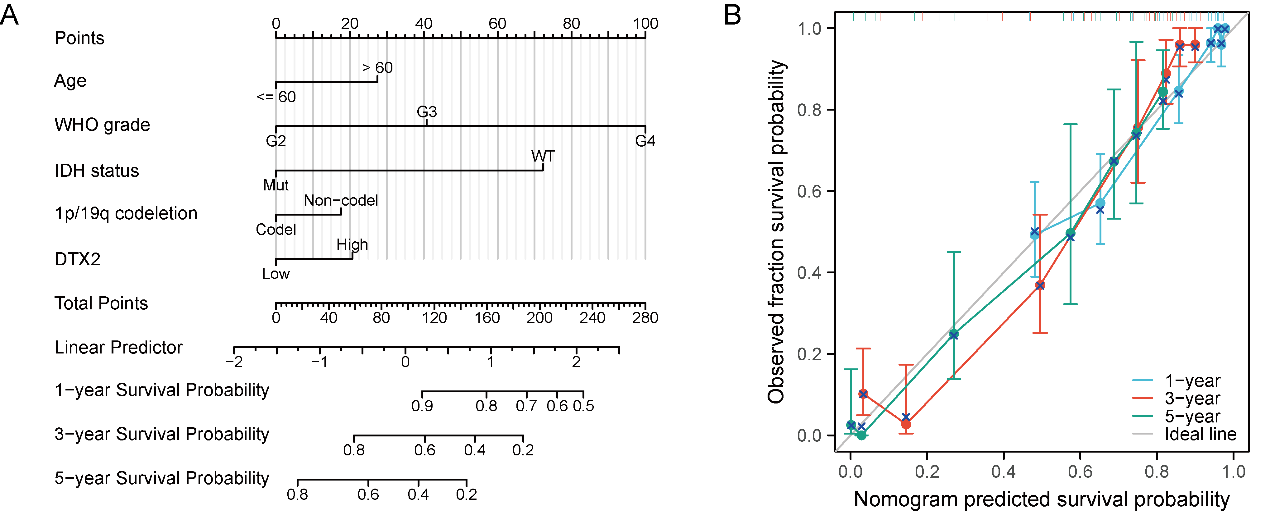
**

**Figure S2. Analysis of the survival value of DTX2 in glioma.** (A) Construction of a prognostic nomogram for glioma survival. The higher the score, the lower the patient's survival rate is likely to be. (B) The calibration curves indicated that the prognostic model had more accurate predictive performance at 1,3,5 years.

**Figure S3**

**
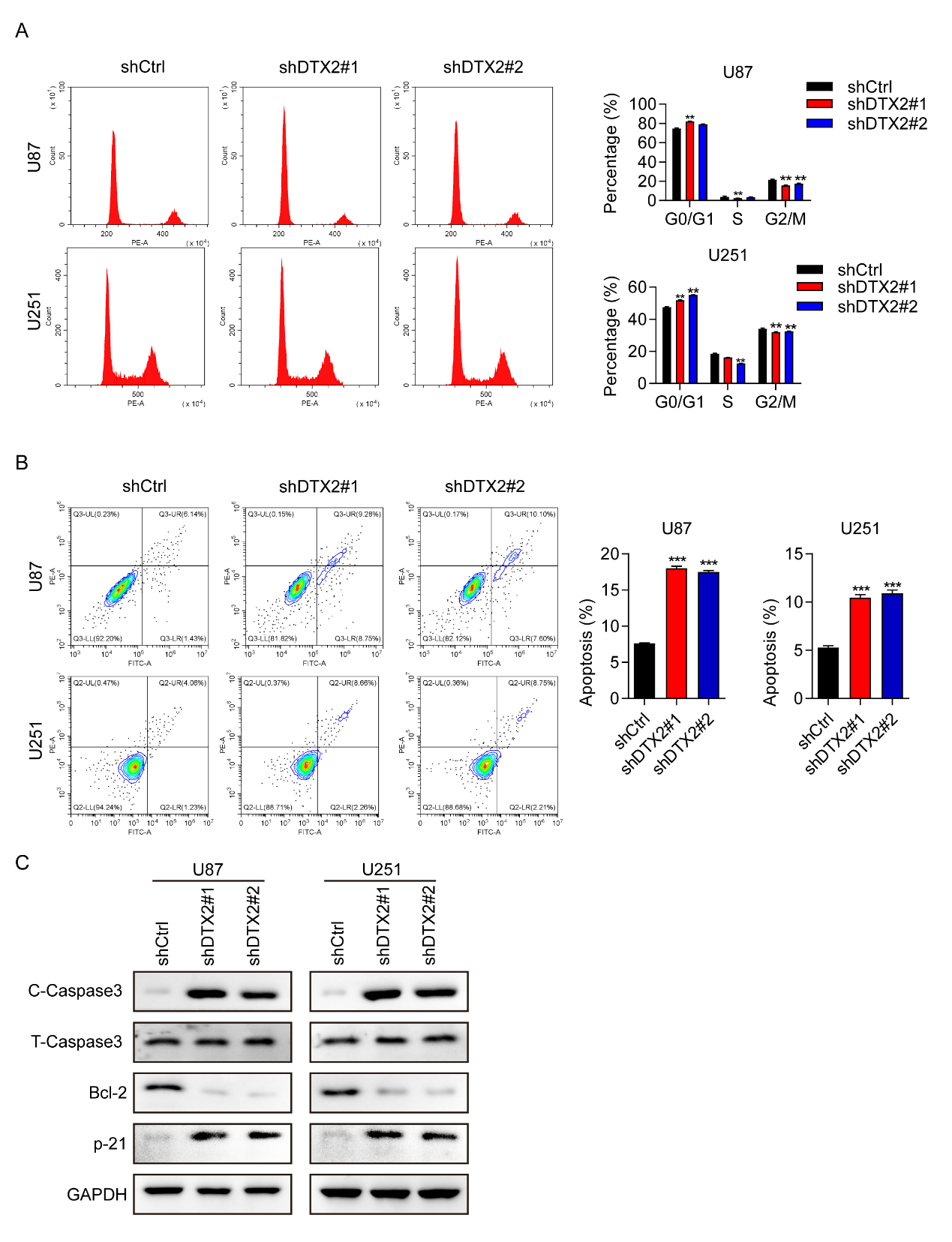
**

**Figure S3.** **The effect of DTX2knock down on cell cycle and cell apoptosis in glioma.** (A) Cell cycle was determined in glioma cells after DTX2 knockdown in U87 and U51 cells using flow cytometry. (***P* < 0.01). (B) Cell apoptosis was determined in glioma cells after DTX2 knockdown or overexpression using flow cytometry. (****P* < 0.001). (C) The expression of apoptosis (c-caspase 3 and t-caspase3) and cell cycle protein (p21) in glioma cells after DTX2 knockdown and overexpression was investigated by western blotting. (***P* < 0.01)
